# Patient-Centered Data Home: A Path Towards National Interoperability

**DOI:** 10.3389/fdgth.2022.887015

**Published:** 2022-07-13

**Authors:** Karmen S. Williams, Shaun J. Grannis

**Affiliations:** ^1^Department of Health Policy and Management, Population Health Informatics, City University of New York, New York, NY, United States; ^2^Center for Biomedical Informatics, Regenstrief Institute, Indianapolis, IN, United States; ^3^Richard M. Fairbanks School of Public Health, Indiana University, Indianapolis, IN, United States; ^4^Indiana University School of Medicine, Indiana University, Indianapolis, IN, United States

**Keywords:** patient-centered data home, health information exchange organizations, overlap analysis, patient matching, validation

## Abstract

**Objective:**

National interoperability is an agenda that has gained momentum in health care. Although several attempts to reach national interoperability, an alerting system through interconnected network of Health Information Exchange (HIE) organizations, Patient-Centered Data Home (PCDH), has seen preliminary success. The aim was to characterize the PCDH initiative through the Indiana Health Information Exchange's participation in the Heartland Region Pilot, which includes HIEs in Indiana, Ohio, Michigan, Kentucky, and Tennessee.

**Materials and Methods:**

Admission, Discharge, and Transfer (ADT) transactions were collected between December 2016 and December 2017 among the seven HIEs in the Heartland Region. ADTs were parsed and summarized. Overlap analyses and patient matching software were used to characterize the PCDH patients. R software and Microsoft Excel were used to populate descriptive statistics and visualization.

**Results:**

Approximately 1.5 million ADT transactions were captured. Majority of patients were female, ages 56–75 years, and were outpatient visits. Top noted reasons for visit were labs, screening, and abdominal pain. Based on the overlap analysis, Eastern Tennessee HIE was the only HIE with no duplicate service areas. An estimated 80 percent of the records were able to be matched with other records.

**Discussion:**

The high volume of exchange in the Heartland Region Pilot established that PCDH is practical and feasible to exchange data. PCDH has the posture to build better comprehensive medical histories and continuity of care in real time.

**Conclusion:**

The value of the data gained extends beyond clinical practitioners to public health workforce for improved interventions, increased surveillance, and greater awareness of gaps in health for needs assessments. This existing interconnection of HIEs has an opportunity to be a sustainable path toward national interoperability.

## Introduction

National interoperability, the ability for systems and software to exchange information across the United States is a goal sought by various stakeholders in the health care field. The dramatic increase in the amounts of data available due to electronic health records and health information technology adoption, creates opportunities for greater access to patient data, availability of more comprehensive health records, and improved care coordination among various providers (1–3). Despite the rise, health information exchange is still siloed in health systems, hospitals, and within state lines. The Patient Centered Data Home (PCDH) is an initiative to connect these silos using a network of health information exchange (HIE) organizations. This study aimed to characterize the PCDH initiative through the lens of the Heartland Region pilot.

Patient-centered care seeks to strengthen patient-clinician relationships, promote communication, and empower patient involvement in health care (4). To reach effective patient-centered care, access to accurate and timely information for informed health decision-making is pertinent (5). In addition to the lack of information at the beginning of a visit, studies indicate only half of patients in the emergency rooms receive timely follow-up after discharge. Patients who do not receive follow-up care are more likely to have worsening conditions and return to the emergency department (6–13). Additionally, because all health care is not local, patches in patient information are inadequate for proper patient-centered care. The wide adoption of electronic health records improves the availability of patient health information to inform decision-making during a patient visit. Follow-up care coordination also benefits from this adoption (2). Although advances in governance and patient consent have improved the exchange of EHR data, it is still frequently siloed within hospitals and health systems.

Silos of health information exchange are characterized as data systems that do not exchange data with other similar systems (14). Challenges in maturity such as, the lack of adequate infrastructure, electronic health record data integration, technical support, security features, and training for staff, add to the disconnection and lack of exchange between health systems (15). The lack of supportive infrastructure through HIEs is especially elevated in rural areas (16). Lack of clarity in the return on investment, leadership investment, readiness and ability of HIEs, and privacy and security concerns also hinders the exchange of health information (15, 17).

Health information exchange on a global scale was examined in China, England, India, Scotland, Switzerland, and United States. Most of the countries had a level of implementation of electronic records, exchange of those records were fully or partially implemented at some levels (19). The authors indicated that the implementation of health information exchange needs a desire to optimize the quality and outcomes of healthcare with relevant, timely, and accurate patient information at the point of care. As these countries are mostly developed countries, more research is needed to characterize health information exchange in developing countries. The authors also concluded that supportive structures, such as economic, political, and cultural, are needed to combat challenges for health information exchange (19).

Challenges with health information exchange has seen relief through Health Information Exchange (HIEs) organizations that serve as secure, comprehensive storage and exchange centers for patient data from various health and community organizations. HIEs use a variety of technological approaches to improve provider access to patient information both collected and maintained by other organizations. Exchange of information by HIEs has been timely, comprehensive, and addresses threats to quality, safety, and efficiency (20). HIE benefits include reduced duplicate procedures, improved patient safety, and lower costs (12, 20). Regional HIEs expanded the scope of exchanging health data electronically but are still limited to regions and states.

In order to facilitate electronic health exchange, accurate patient matching is necessary. Lack of consistent evidence based guidelines for patient matching has been a continuous barrier to improving interoperability (21). Patient matching algorithms use patient demographics and patient identifiers to link records (22–25). Independent entity agreement, cohesion on data governance, and the appropriate process of patient matching itself has been a source of challenge. Nicknames, married names, security, merging and unmerging data, updates from hospitals and HIES, and strengths and limitations to understand each HIE, can slow the patient matching process. Patient matching has the potential to enhance patient care and ultimately patient satisfaction, reduce costs from duplicated testing, inform innovation through patient outcomes, and identify fraudulent activity. However, the challenge with quality and effective patient matching is standardization (25). At a national scale, patient matching can increase continuity of care among multiple providers.

In 2009, Healthbridge, a Health Information Exchange Network in Cincinnati, Ohio, and the Indiana Health Information Exchange (IHIE), in Indianapolis, Indiana, developed an “electronic postman” approach reducing the use of fax to exchange clinical health data between Indiana and Ohio (26). In 2012, The Sequoia Project began management of a nationwide health information exchange, eHealth Exchange, which focused on governmental agencies' exchange of health information (27). Also directed by The Sequoia Project, Carequality expanded interoperability nationwide including various non-governmental agencies, such as private care providers, hospitals, and clinics (28). Recently, the National Coordinator for Health Information Technology began the Trusted Exchange Framework and Common Agreement to link electronic data sharing islands (29). Vendors, such as EPIC and Cerner, have enlarged the scope of health data exchange beyond their systems. Although there is successful data exchange on these larger known scales, there are many data exchange initiatives that operate in silos without connection to others and are not widely known.

The purpose of this study was to characterize the PCDH initiative from the perspective of Indiana Health Information Exchange's participation in the Heartland Region Pilot (described below). This manuscript describes one approach toward national interoperability using an interconnected network of HIEs to exchange patient health information across state lines while accommodating individual HIE governance and standards. Although in its beginning stages, PCDH has shown operating potential as a pathway toward national interoperability. This manuscript explored the exchange and data generated through this beginning process. Although there has been some discussion on PCDH, this is the first published evaluation of the PCDH initiative. Additionally, conducting an overlap analysis and patient matching to our characterization of this data are unique and key features of this study.

The structure of the remainder of this paper includes an origin of PCDH and the Heartland Pilot, the process of PCDH, explanations and results of data collection, zip code validation, overlap analysis, chief complaint classification, patient matching, and analyses of these data.

## Methods

**Figure 1:**
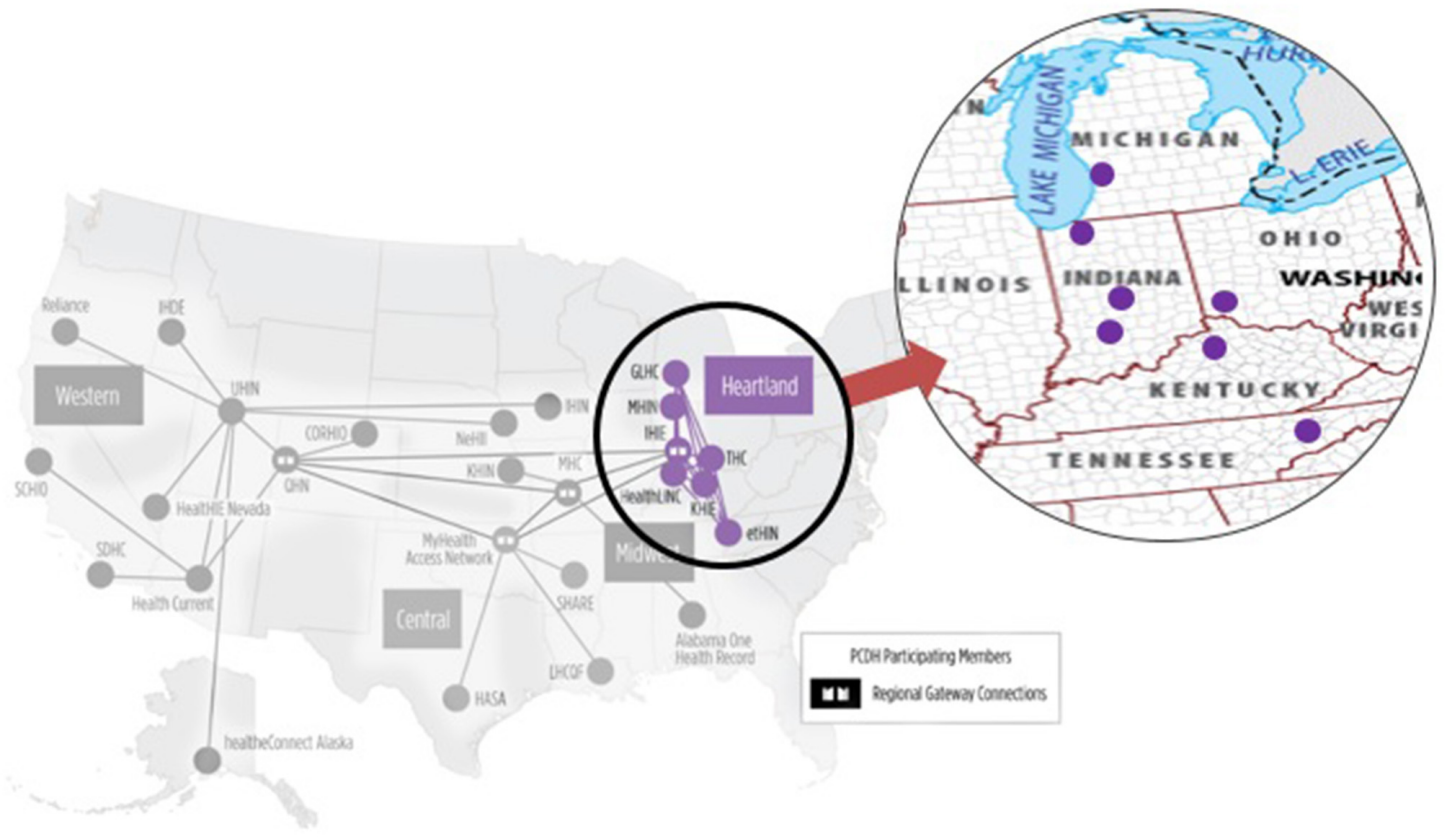
Heartland region of patient-centered data home (18).

The Strategic Health Information Exchange Collaborative (SHIEC) “is a national collaborative representing health information exchanges (HIEs) (18)”. With over 70 HIEs, SHIEC covers more than 200 million people across the United States (18). SHIEC having this coverage, implemented three PCDH pilots: Western Region, Central Region, and Heartland Region. The Western Pilot includes Arizona Health-e Connection, Quality Health Network in Western Colorado, and Utah Health Information Network. The Central Hub Pilot includes MyHealth Access Network in Oklahoma and Texas, and Arkansas State Health Alliance for Records Exchange. The Heartland Region includes 7 HIEs in 5 states: East Tennessee Health Information Network in Knoxville, Tennessee; Great Lakes Health Connect in Grand Rapids, Michigan; HealthLINC in Bloomington, Indiana; Indiana Health Information Exchange in Indianapolis, Indiana; Kentucky Health Information Exchange in Frankfort, Kentucky; Michiana Health Information Network in South Bend, Indiana; and The Health Collaborative in Cincinnati, Ohio (Figure 1). The pilot began in September 2016, by supporting alerting and sharing summary information among these seven HIEs. In January 2018, all three regions were connected, demonstrating a next step toward national interoperability (18).

- **etHIN**—East Tennessee Health Information Network—Knoxville, TN
- **GLHC**—Great Lakes Health Connect—Grand Rapids, MI
- **IHIE—**Indiana Health Information Exchange—Indianapolis, IN
- **HL—**HealthLINC (HL)—Bloomington, IN
- **KHIE—**Kentucky Health Information Exchange—Frankfort, KY
- **MHIN**—Michiana Health Information Network—South Bend, IN
- **THC—**The Health Collaborative—Cincinnati, OH.

**Figure 2:**
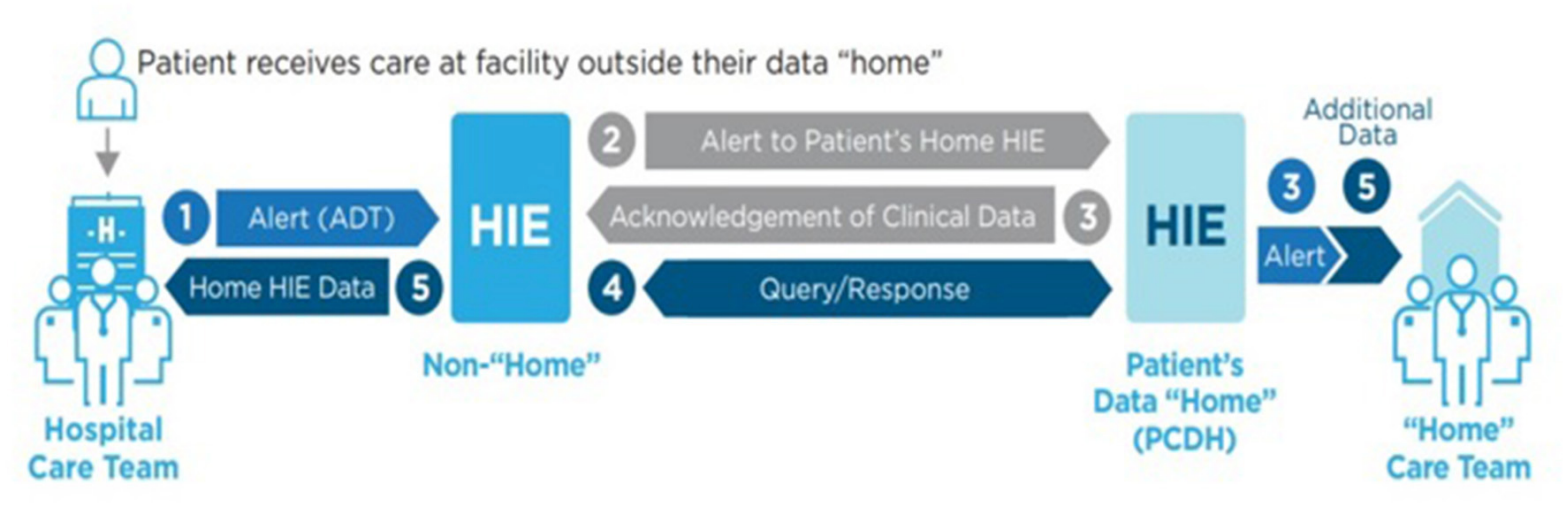
Patient-centered data home process (18).

In the PCDH process, the HIE that services the patient's residential zip code is designated as the storage center and “home” HIE. PCDH's goal is to store a comprehensive, longitudinal patient record in the “home” HIE; in where all data sent and received becomes part of the home record. At the beginning of the PCDH pilot, zip codes from the HIE service area were shared among the HIEs in the Heartland region. The PCDH process is triggered when a patient visits another medical facility not in their “home” HIE service area, called an “away” HIE (Figure 2). The facility in the “away” HIE queries the “home” HIE requesting access to available patient information. If available, the “home” HIE generates an HL7 version 2, Admission, Discharge, Transfer (ADT) message that contains pertinent medical information. Once the encounter is completed, the “away” HIE sends a summary ADT to alert the home HIE that there are new records available. The PCDH process allows for comprehensive medical histories to follow a patient wherever treatment is sought. In the future, the care documents will be stored in the home HIE for care providers to see and select as appropriate.

The study received protocol approval from the Indiana University Institutional Review Board and the Indiana Health Information Exchange securely released the data for analysis. The sample for this exploratory study was collected December 2016 through December 2017. Using HL7 version 2 ADT messages, summary data was both sent and received by the Heartland Region HIEs. The HL7 version 2 messages were de-duplicated to identify unique encounters for the PCDH initiative. Once de-duplicated, specific fields were parsed for analysis: sending facility, receiving facility, message date, patient date of birth (year, month, day), patient gender, patient social security number, patient state of residence, chief complaint or reason for visit, and patient class (referred to as visit type). Transactions missing the parsed fields were excluded from analysis due to the inability to definitively identify them as an encounter.

**Figure 3:**
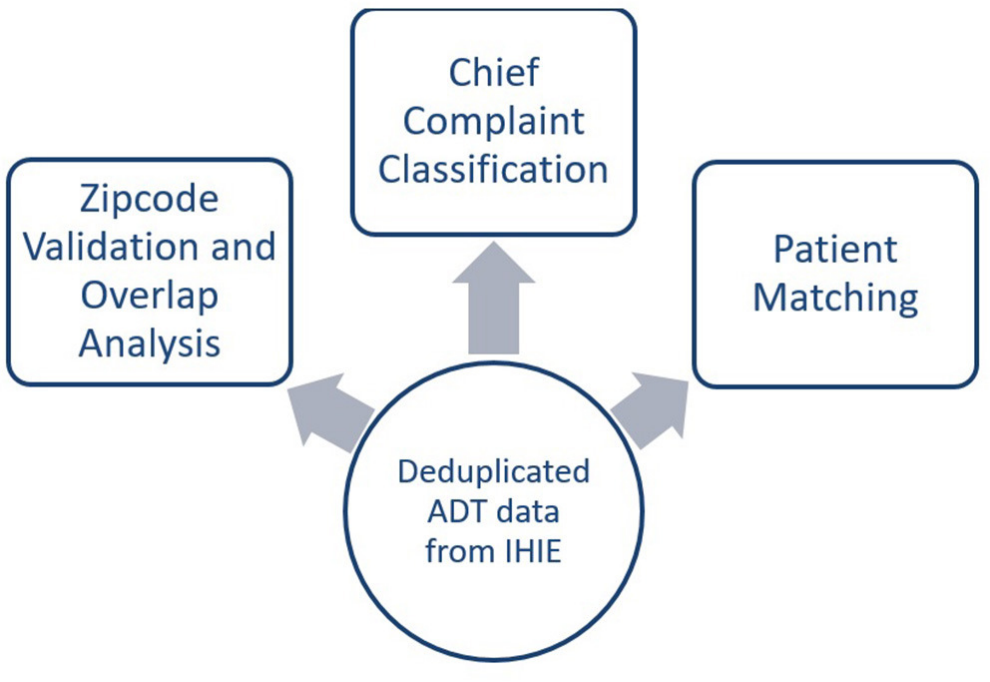
Methodology steps of PCDH study.

Using the deduplicated and parsed ADT data, a zip code validation and overlap analysis was conducted to determine the coverage areas of the HIEs. A chief complaint classification to determine the reasons for the visit, and deterministic probabilistic patient matching were applied to the data for further characterization of the data (Figure 3). Further explanation of each can be found below.

### Zip Code Validation

The Heartland Region HIEs compiled a list of zip codes that represented each HIE service area. At the beginning of the PCDH pilot, most of the HIEs had a 5 percent threshold of patients in a zip code for that zip code to be associated with that HIE. In April and May of 2017, the threshold was changed to 10 percent. The volume of exchange presented in the results reflect these changes. To ensure that only PCDH pilot data was analyzed, a zip code validation was performed. This validation involved matching zip codes of the sending and receiving facilities against the HIE provided zip code tables. If the ADT messages were not in compliance with the PCDH pilot zip codes, those encounters were excluded from analysis.

### Overlap Analysis

An analysis was conducted to examine the overlap of each HIE service area. The HIE zip code service areas were provided by IHIE to analyze the coverage and percent of overlap shared in the Heartland Region. Using Microsoft Excel, duplicate zip codes were identified, and proportions calculated for each HIE. Formulas were applied to detect duplicates on the entire zip code data set. After adjustments to the formulas and calculations in the spreadsheets, color-coded maps and visualizations were created using Microsoft Power BI (Microsoft, v.2, 2018) (30).

**Figure 4:**
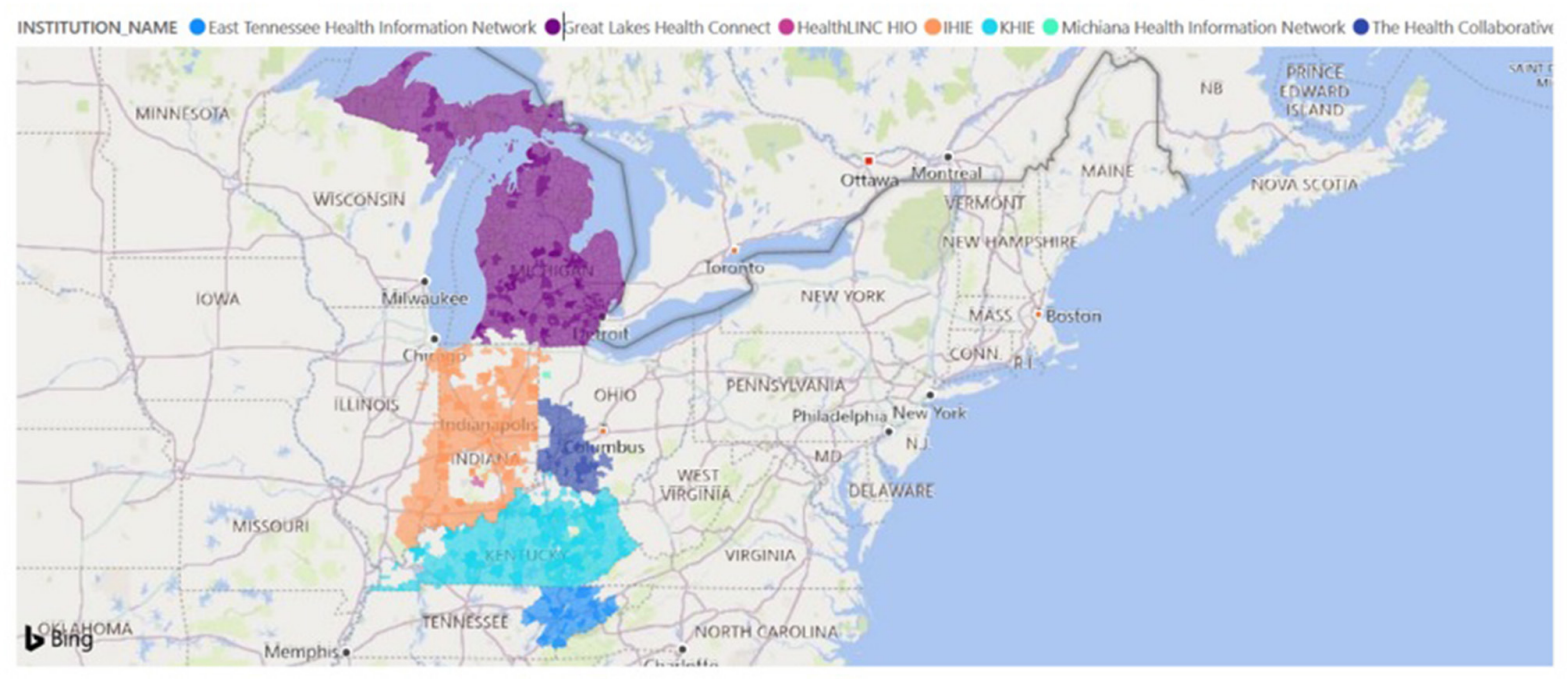
Overlap analysis of heartland region HIE zip codes.

After the initial zip code overlap analysis was conducted on the HIEs in the Heartland Region, the validated PCDH zip codes were cleaned and extracted from the data and added to the Microsoft Excel spreadsheet. The validated PCDH zip codes were assigned to their respective HIEs and another overlap analysis was conducted to examine the coverage areas. Duplicates were identified and removed from the final analysis (Figure 4). Additionally, non-reported zip codes were located and corrected. Maps and color-coded visualizations were created in Microsoft Power BI.

We determined that the Heartland Region HIEs had 4,062 unique zip codes. Eastern Tennessee HIN with zero shared zip codes, had 100% non-overlapping coverage of their service area. The majority of Great Lakes Health Connect, Kentucky Health Information Exchange, and The Health Collaborative were unshared zip codes. As the largest HIE in the region, IHIE had the most unshared zip codes and received the most ADTs (Table 1). Although Great Lakes Health Connect had the second most zip codes, there were less ADTs than 3 of the HIEs. With 394 zip codes in the region, Michiana Health Information Network had the second highest frequency of ADTs.

**Table 1:** Unshared zip code counts and ADT messages within that zip code in the PCDH.

**Unshared PCDH Zip Codes**			
**HIEs**	**Number of** **Zip Codes**	**ADTs in Zip Code**	
		***n***	**%**
Eastern tennessee health information network	84	320	0.16%
Great lakes health connect	572	3,172	1.55%
HealthLINC	1	39	0.02%
Indiana health information exchange	700	181,083	88.39%
Michiana health information network	394	15,351	7.49%
Kentucky health information exchange	2	18	0.01%
The health collaborative	190	4,874	2.38%
Total	1,943	204,857	

Table 2 shows consistencies with the tables above; all of the Eastern Tennessee HIE zip codes were covered uniquely by Eastern Tennessee Health Information Network. Also, Michiana and HealthLINC were nearly completely duplicated coverage. Of the zip codes that overlap, Indiana Health Information Exchange and Michiana Health Information Network had the greatest proportion of overlap (26.7%).

**Table 2:** Shared zip code counts and ADT messages within that zip code in the PCDH.

**Shared PCDH Zip codes**			
**HIEs**	**Number of** **Zip codes**	**% Overlap**	**ADTs in** **Zip Code**
Great lakes health connect and Indiana health information exchange	2	0.5%	870
Great lakes health connect and Indiana health Information exchange and Michiana health information network	5	1.3%	5,197
Great lakes health connect and Michiana health information network	17	4.3%	1,773
HealthLINC and Indiana health information exchange	76	19.1%	232,499
Indiana health information exchange and Michiana health information network	106	26.7%	673,045
Indiana health information exchange and the health collaborative	53	13.4%	163,087
Indiana health information exchange and Kentucky health information exchange	31	7.8%	209,535
Indiana health information exchange and Kentucky health information exchange and the health collaborative	31	7.8%	340
Kentucky health information exchange and the health collaborative	76	19.1%	1,131
TOTAL	397		1,287,477

### Chief Complaint Classification

A chief complaint, as defined by the Current Procedural Terminology (CPT) codebook, is a “concise statement describing the symptom, problem, condition, diagnosis, or other factor that is the reason for the encounter, usually state in the patient's words (31).” Each ADT message contained a column for chief complaints. The validated ADT messages from each HIE were merged into a single file via R programming (v.3.5.3) for further analysis (32). We used an R-based natural language processing package, *NLP*, to process the chief complaints. The chief complaints were sorted by frequency. We manually standardized by merging entries with x, blank, or other characters as blank; codes and non-recognizable words as unknown; and linking the same words for similar complaints and procedures (i.e., lt leg pain, left leg pain, l leg pain = left leg pain). We deduplicated the spreadsheet and re-sorted by frequency.

### Patient Matching

After validating the transactions, we identified unique PCDH encounters. This is necessary because clinical systems often send multiple electronic registration transactions for the same clinical encounter: an initial transaction indicating the beginning of an encounter (ADT^∧^A04) and subsequent transactions to update or add information not gathered during the initial intake (ADT^∧^A08). Based on our longstanding operational experience and prior data analyses, we used data elements including person, place and time to establish unique emergency department encounters (24, 25). The specific fields included (1) healthcare institution (HL7 MSH-4), (2) encounter date (HL7 PV1–44), and (3) medical record number (HL7 PID-3). Transactions missing any of these fields could not be definitively and uniquely identified as an encounter and were excluded from the analysis.

We next identified unique patients among the set of unique encounters. This task is identical to the process of finding duplicate patients in a patient registry: we linked the dataset containing all unique encounters to itself. To accomplish this we used an open-source deterministic record linkage software package. Deterministic matching generally exhibits high sensitivity than probabilistic. We sought to maximize matching specificity for this analysis. To link patients we used various combinations of patient demographics, including social security number, last and first name, gender, date of birth, telephone number, and zip code as determined by the record linkage software. In this manner all encounters belonging to the same patient were linked, forming a “patient group.” We randomly assigned a unique global patient identifier to each patient group. Each unique PCDH encounter was assigned the appropriate global patient identifier (24, 25). To check matching accuracy, we manually checked randomly selected records.

### Data Analysis

We generated summary statistics in the final data set containing validated and unique encounters using R Software and Microsoft Excel. Microsoft Excel and Microsoft Power BI were used to visualize the volume of exchange among the Heartland Pilot, the overlapping of service areas, and characteristics of the population within the PCDH pilot sample.

## Results

The results indicate a robust exchange rate between December 2016 and 2017. The findings show the messages sent and received between the Heartland Region, and demographics, such as, ages, sex, facility types, chief complaints, and patient matching.

The PCDH Heartland Region pilot generated 1,492,367 ADT messages. IHIE sent an estimated 805,994 ADT messages and received 686,373 ADT messages. Table 3 indicates the direction and volume of ADT messages sent and received by IHIE during the Heartland Region PCDH pilot. The majority of the messages were sent from MHIN, KHIE, and HL from IHIE, while IHIE received the most messages from MHIN, HL, and THC.

**Table 3:** Volume of ADT messages sent and received to the Indiana Health Information Exchange during the Heartland Region PCDH Pilot (December 2016–December 2017).

**Heartland Region HIEs**	**RECEIVED** **by IHIE**	**SENT** **by IHIE**
Eastern Tennessee health information network	320	211
Great lakes health connect	8,473	7,278
HealthLINC	162,476	87,694
Kentucky health information exchange	79,904	306,735
Michiana health information network	300,444	372,981
The health collaborative	134,756	31,095

### Demographics

**Figure 5:**
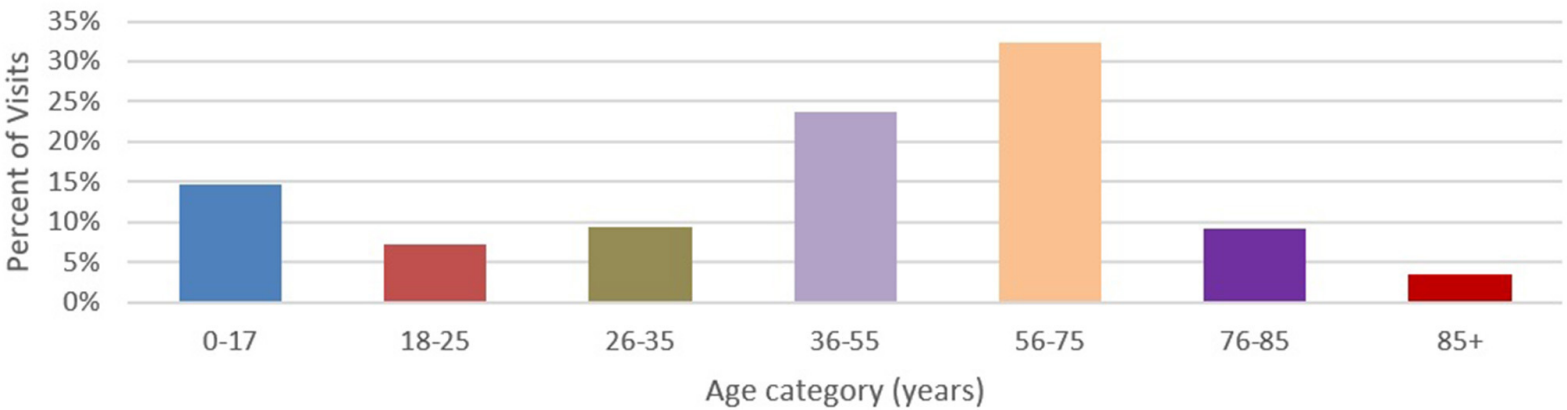
Age categories by percent of exchange (*n* = 1,492,109).

Approximately 58 percent of the sample were females and 42% males. Ages 56–75 years (32.3%) were the most represented in the sample, followed by 36–55 years (23.7%) and 0–17 years (14.7%) (Figure 5).

An estimated 82% of patients were residents of Indiana, followed by Kentucky (14%) and Ohio (2%). Over half of the messages were from outpatient (60%) areas. Emergency departments (27%), other departments (not included in outpatient or emergency department codes), and inpatient (3%) made up the remaining visit types.

Out of the 1,492,367 chief complaints, over half were blank or undecipherable (54%). Among the chief complaints with entries, the top 5 chief complaints or reason for visit were laboratory orders, screening, abdominal pain, chest pain, and difficulty breathing or shortness of breath.

### Patient Matching

Five deterministic blocking schemes were conducted on the PCDH dataset. The sets were restricted to record pairs with either a different Sending Fax or different Visit Date to deduplicate the records for the same visit. The records were grouped into pairs based on the matches. Combining the results of all 5 blocking schemes, there are 5,040,537 record pairs grouped into 271,299 patient groups. These group counts represent the distinct patients with matched pairs, not the total distinct patients. Each group has a median of 3 records per group. From the starting dataset of 1,492,372 records, 1,190,513 records (80%) are in at least one record pair (Table 4). This indicated that the algorithm was sufficient to match most of the records to the patients in the pilot records.

**Table 4:** Deterministic patient matching results.

**Total records = 1,492,372**	**SSN + DB +** **MB + YB**	**SSN + FN +** **MB + YB**	**SSN + LN** **+ DB**	**SSN + LN** **+ FN**	**LN + FN + G** **+ DB + MB + YB**	**LN + FN + ADR** **+ ZIP + YB**	**Combined**
Matched record pairs total	4,097,247	4,082,686	4,076,314	4,065,206	5,024,822	4,164,494	5,063,821
Matched record pairs, excluding duplicate encounters	4,078,595	4,064,152	4,057,774	4,046,748	5,001,807	4,145,573	5,040,537
Matched patient groups	208,159	208,039	207,943	207,898	271,121	258,921	271,299
Records in at least one matched pair	922,657	920,644	919,825	918,409	1,185,358	1,057,296	1,190,513
% of Records in at least one matched pair	61.8%	61.7%	61.6%	61.5%	79.4%	70.8%	79.8%

Through these findings there is an indication of value of the PCDH as a viable solution to cross-state interoperability and better continuity of care across patient care teams.

## Discussion

In characterizing the PCDH initiative from the perspective of Indiana Health Information Exchange's (IHIE) participation, findings indicate that the PCDH framework is practical and feasible to exchange data. Building upon past attempts and knowledge from failed initiatives, the PCDH initiative has found a path to make national interoperability actually work.

### Overlap Analysis

As indicated by the findings, six of the seven Heartland Region HIEs share patients from another HIE in the region. Thirty six of the zip codes are shared among 3 of the HIEs in the region, sharing approximately 5,500 ADT messages during the pilot. This level of sharing is unique among the Heartland Region pilot, as the Central and Western Region HIEs were spread father apart among the region. Determining a way to reduce duplicated efforts could provide greater efficiency and sharing practices among the Heartland Region HIEs.

### Rate of Exchange

Due to the geographic closeness of the Heartland Region HIEs, we expected to see overlap of service areas. Also, because IHIE is one of the largest HIEs in the country and 84 percent of the ADT messages were for Indiana state residents, there was confidence that that most of the HIEs sent their messages to IHIE. Since the overlap analysis showed a mutual coverage of 80% of Michiana Health Information Network's zip codes, the volume of exchange between IHIE and Michiana Health Information Network was confirmed. At the beginning of the PCDH pilot, there were technical issues and delayed exchange which have impact on the numbers. However, consistent with the overlap analysis and expectations, the HIEs furthest away from IHIE, GLHC in Michigan and ETHIN in Tennessee, had lower volumes of exchange.

### Gender and Age

Consistent with other findings in the Heartland Region, more females are represented in HIEs than males. This is also congruent with the national standard. However, with age groups, there is much inconsistency. This could be due to population differences in the Heartland Region or the ability of certain ages to be mobile than others. A deeper analysis and further study could help to illuminate the differences between age demographics and seeking health care in other areas, states, and regions.

### Chief Complaints

Consistent with findings in the outpatient settings of the Heartland Region area, laboratory orders and screening were most frequently mentioned chief complaint. However, abdominal pain was the third instead of lower back pain. Also consistent with emergency department settings in the Heartland Region, abdominal pain, chest pain, and falls were among the most commonly indicated reason for visit. These findings indicate that the PCDH is exchanging quality, meaningful data that closely represents the population in the region.

### Patient Matching

Over 271,000 patients were matched into groups using deterministic patient matching. This indicated that nearly 80 percent of the records in the PCDH pilot were matched to other records in the study. Researchers found that better standardization of the data for last names and addresses could improve this match rate even further for better care decision-making and delivery (25). Further exploring the characteristics of the matched patient groups will help to understand the type of patients that are frequently seeking care outside of their home area as well as those who are not. Patient matching bridges the gap for emergency care and primary care to increase the communication and continuity among providers across specialties and the United States. Although there are concerns of privacy and costs, Universal Patient Identifiers are considered a potential solution for achieving true national interoperability, which would allow patient records to be more quickly matched in the PCDH process (33).

### Strengths of Study

The strength of this study was the availability of the large amount of data due to the size of the pilot. The Heartland Region initiating PCDH is that it includes smaller HIEs, which have otherwise been excluded. We postulate that PCDH will lead to better comprehensive medical histories and continuity of care in real time. As HIEs have been helpful during natural disasters with HIEs allowing patient access to their records. PCDH would increase the impact of assistance during these urgent situations (34). Data from and through PCDH can improve population health interventions, increase public health surveillance, assist with health needs assessment requirements, provide greater awareness of health gaps, support decision making for leaders and clinical staff, inform standards and policy, and provide sources of research and social impact.

### Limitations

Although the PCDH initiative is working, there are still changes affecting interoperability. Some of the major challenges include: the difficulty of data, identification, and matching; lack of, multiple, and different standards; various options for reaching interoperability; and aligning leadership and technical expertise. The lack of data standardization provided challenges for matching patient records, even using our deterministic algorithms. As this is a new initiative, misalignment with the leadership picture and the technical details caused delays in both the data collection and analysis process. Additionally, missing data continues to be a major issue as hospitals send HIEs information they are comfortable sending. Patients are allowed to opt-out of data being sent to HIEs, therefore no consent, no data. In emergency situations, there are expedited rules that are validated, unstandardized processes at each HIE.

## Conclusion

The value of this data extends beyond clinical practitioners to the public health workforce for improved interventions, increased surveillance, and greater awareness of gaps in health for needs assessments. We expect that SHIEC's existing interconnection of HIEs will provide a sustainable path toward national interoperability. In January 2018, all three regions were connected, demonstrating a next step toward national interoperability (35). In June of 2020, North Carolina celebrated being one of the newest HIEs to join the PCDH national initiative in the Midwest region, which includes Alabama, Missouri, Kansas, and Wisconsin. Between April 2020 and June 2020 the HIE exchanged over 11,000 ADT notifications with “18 HIEs for out-of-state patient visits” (36).

PCDH uses a “bottom–up” approach to interoperability. Future examination of the impact of PCDH and use in the clinical setting as well as for population health will be necessary. Future work research could include integration of other systems, such as those with social determinants of health features, studying other pilots across the nation, including various medical, dental, and pharmaceutical areas, and ways to continue to improve the process toward sustainable methods of national interoperability.

At the completed phases, PCDH plans benefits of centralization through a national ADT alerting and identifying assurance, accurate gap analyses, precise quality measures, option for centralized patient consent management, and patient access to their entire record in one location. With low costs and scalability PCDH seeks to continue to expand and include all levels of HIEs. Each HIE's policies, technology and values will be honored by preserved governance, sustained management processes, honored data use agreements, maintained privacy and consent models, unchanged business models, and conserved technical architecture. During this study, ADTs were the only alerts being transferred but the process had begun to send and receive continuity of care documents for a more comprehensive record in individuals' home record.

The Heartland Region found success in having a peer-to-peer networking, having one gateway hub in the region to route to other gateway hubs, and utilizing a tiered process to achieve national interoperability. PCDH has moved away from the idea that national interoperability will happen one way only and accepts the possibility of all ways. Despite challenges, PCDH has found actual success toward national interoperability. Currently, SHEIC has reported 45 HIEs in PCDH, with over 177 million total event notifications exchanged by HIEs (37). Future research should include an evaluation of the current PCDH and outcomes, and potential implications for multinational interoperability.

## Data Availability Statement

The datasets presented in this article are not readily available because Data was given by Indiana Health Information Exchange and would need permission to access. Requests to access the datasets should be directed to https://www.ihie.org/contact-us/.

## Author Contributions

KW created the first draft of the manuscript. Both authors contributed to manuscript revision, read, and approved the submitted version. Both authors contributed to the conception, design of the study, and performed the statistical analyses.

## Funding

KW was supported by a training grant (Award Number T15NLM012502) from the National Library of Medicine of the National Institutes of Health.

## Author Disclaimer

The content is solely the responsibility of the authors and does not necessarily represent the official views of the CDC, NIH or HHS.

## Conflict of Interest

The authors declare that the research was conducted in the absence of any commercial or financial relationships that could be construed as a potential conflict of interest.

## Publisher's Note

All claims expressed in this article are solely those of the authors and do not necessarily represent those of their affiliated organizations, or those of the publisher, the editors and the reviewers. Any product that may be evaluated in this article, or claim that may be made by its manufacturer, is not guaranteed or endorsed by the publisher.

## Abbreviations

HIE: Health Information Exchange organization
PCDH: Patient Centered Data Home
IHIE: Indiana Health Information Exchange
ADT: Admissions, Discharge, Transfer.

## References

[1] American Medical Informatics Association. Redefining Our Picture of Health: Towards a Person-Centered Integrated Care, Research, Wellness, and Community Ecosystem [White Paper] (2017).

[2] Williams KS Shah G Leider JP Gupta A. Overcoming barriers to experience benefits: a qualitative analysis of electronic health records and health information exchange implementation in local health departments. eGEMS. (2017) 5:18. 10.5334/egems.216 29881738 PMC5983057

[3] Cross DA Stevens MA Spivack SB Murray GF Rodriguez HP Lewis VA. Survey of information exchange and advanced use of other health information technology in primary care settings: capabilities in and outside of the safety net. Med Care. (2022) 60:140–8. 10.1097/MLR.0000000000001673 35030563 PMC8966676

[4] Epstein R Street R. The values and value of patient-centered care. Ann Fam Med. (2011) 9:100–3. 10.1370/afm.1239 21403134 PMC3056855

[5] Horahan K Morchel H Raheem M Stevens L. Electronic health records access during a disaster. Online J Public Health Inform. (2014) 5:e232. 10.5210/ojphi.v5i3.4826 24683443 PMC3959913

[6] Chen J Singer E. Primary care follow-up after emergency department visits for routine complaints: What primary care physicians prefer and what emergency department physicians currently recommend. Pediatr Emerg Care. (2016) 32:371–6. 10.1097/PEC.0000000000000314 25695845

[7] Budryk Z. Care Coordination Challenges: Poor Communication, Tracking Patient Populations. Fierce Healthcare Available online at: https://wwwfiercehealthcarecom/special-report/care-coordination-challenges-poor-communication-tracking-patient-populations (accessed February 21, 2022).

[8] Agency for Healthcare Research Quality. Prospects for Care Coordination Measurement Using Electronic Data Sources. Available online at: https://www.ahrq.gov/research/findings/final-reports/prospectscare/prospects1.html (accessed February 21, 2022).

[9] Jackson C Shahsahebi M Wedlake T DuBard CA. Timeliness of outpatient follow-up: an evidence-based approach for planning after hospital discharge. Ann Fam Med. (2015) 13:115–22. 10.1370/afm.1753 25755032 PMC4369604

[10] Sinha S Seirup J Carmel A. Early primary care follow-up after ED and hospital discharge—does it affect readmissions? Hosp Prac. (2017) 45:51–7. 10.1080/21548331.2017.1283935 28095063

[11] DeLia D Tong J Gaboda D Casalino LP. Post-discharge follow-up visits and hospital utilization by Medicare patients, 2007–2010. Medicare Medicaid Res Rev. (2014) 4:mmrr.004.02.a01. 10.5600/mmrr.004.02.a01 24949226 PMC4062381

[12] Fecher K McCarthy L Porreca D Yaraghi N. Assessing the Benefits of Integrating Health Information Exchange Services into the Medical Practices' Workflow. Inform Sys Frontiers. (2020) 23:599–605. 10.1007/s10796-019-09979-x

[13] Han X Lowry TY Loo GT Rabin EJ Grinspan ZM Kern LM Kuperman GJ Shapiro JS. Expanding health information exchange improves identification of frequent emergency department users. Ann Emerg Med. (2019) 73:172–9. 10.1016/j.annemergmed.2018.07.024 30236418 PMC12945391

[14] Miller AR Tucker C. Health information exchange, system size and information silos. J Health Econ. (2014) 33:28–42. 10.1016/j.jhealeco.2013.10.004 24246484

[15] Chandrasekaran R Sankaranarayanan B Pendergrass J. Unfulfilled promises of health information exchange: what inhibits ambulatory clinics from electronically sharing health information? Intl J Med Informatics. (2021) 149:104418. 10.1016/j.ijmedinf.2021.104418 33640839

[16] Nakayama M Inoue R Miyata S Shimizu H. Health information exchange between specialists and general practitioners benefits rural patients. Appl Clin Inform. (2021) 12:564–72. 10.1055/s-0041-1731287 34107543 PMC8189760

[17] Shen N Bernier T Sequeira L Strauss J Pannor Silver M Carter-Langford A. Understanding the patient privacy perspective on health information exchange: a systematic review. Intl J Med Inform. (2019) (125):1–12. 10.1016/j.ijmedinf.2019.01.014 30914173

[18] *Patient Centered Data Home*. Available online at: https://strategichie.com/patient-centered-data-home/ (accessed February 21, 2022).

[19] Payne TH Lovis C Gutteridge C. Status of health information exchange: a comparison of six countries. J Glob Health. (2019) 9:0204279. 10.7189/jogh.09.020427 31673351 PMC6815656

[20] Misky GJ Wald HL Coleman EA. Post-hospitalization transitions: examining the effects of timing on primary care provider follow-up. J Hosp Med. (2010) 5:392–7. 10.1002/jhm.666 20578046

[21] Menachemi N Rahurkar S Harle C Vest J. The benefits of health information exchange: an updated systematic review. J Am Med Informatics Assoc. (2018) 25:1259–65. 10.1093/jamia/ocy035 29718258 PMC7646861

[22] Pew, Charitable Trust,. Enhanced Patient Matching is |Critical for Achieving Full Promise of Digital Health Records: Accurately Linking Individuals with their Records Essential to Improving Care. Available online at: https://www.pewtrusts.org/-/media/assets/2018/09/healthit_enhanced patientmatching_report_final.pdf (accessed February 2022).

[23] Grannis SJ Overhage JM Hui S McDonald C. Analysis of a probabilistic record linkage technique without human review. AMIA Ann Symp Proc. (2003) 2003:259–63. 14728174 PMC1479910

[24] Finnell JT Overhage JM Grannis S. All health care is not local: an evaluation of the distribution of emergency department care delivered in Indiana. AMIA Annu Symp Proc. 211:409–16. 22195094 PMC3243262

[25] Grannis SJ Xu H Vest JR Kasthurirathne S Bo N Moscovitch B. Evaluating the effect of data standardization and validation on patient matching accuracy. J Am Med Informatics Assoc. (2019) 26:447–56. 10.1093/jamia/ocy191 30848796 PMC7787357

[26] Indiana Health Information Exchange. Docs4Docs. Available online at: https://www.ihie.org/docs4docs/ (accessed February 21, 2022).

[27] The Sequoia Project. Available online at: https://sequoiaproject.org/about-us/ (accessed April 19, 2019).

[28] Carequality. Available online at: https://carequality.org/ (accessed April 19, 2019).

[29] Adler-Midstein J Garg A Zhao W Patel V. A survey of health information exchange organizations in advance of a nationwide connectivity framework. Health Aff. (2021) 40:736–44. 10.1377/hlthaff.2020.01497 33939510

[30] Microsoft Power BI,. Microsoft Power BI. (2018). Available online at: https://powerbi.microsoft.com/en-us/ (accessed January 11, 2019).

[31] American Medical Association (2018). CPT 2019 [Professional Edition]. Chicago, IL. (accessed May 18, 2019).

[32] R Core Team (2013). R: a language and environment for statistical computing. R Foundation for Statistical Computing, Vienna, Austria. Available online at: http://www.R-project.org/ (accessed August 20, 2018).

[33] VanHouten JP Brandt CA. Universal Patient Identification: What it is Why the US Needs it. (2021). Available online at: https://www.healthaffairs.org/do/10.1377/forefront.20210701.888615/ (accessed June 5, 2022).

[34] Leventhal R. The power of data exchange as disaster strikes how HIE leaders have prepared for Hurricane Florence. (2018). Available online at: https://www.hcinnovationgroup.com/interoperability-hie/article/13030707/the-power-of-data-exchange-as-disaster-strikes-how-hie-leaders-have-prepared-for-hurricane-florence (accessed February 21, 2022).

[35] Monica K. SHIEC Patient Centered Data Health Information Exchange Goes Live. (2018). Available online at: https://ehrintelligence.com/news/shiec-patient-centered-data-health- information-exchange-goes-live (accessed November 1, 2018).

[36] Hughes E. NC HealthConnex Goes Live with the National Patient Centered Data Home Network: N.C. Providers Will Now be Alerted When Patients Present at EDs Out of State. (2020). Available online at: https://hiea.nc.gov/news/press-releases/2020/06/10/nc-healthconnex-goes-live-national-patient-centered-data-home (accessed February 21, 2022).

[37] Strategic Health Information Exchange Collaborative. Nationwide interoperability enabled by the Patient Centered Data Home. (2022). Available online at: https://strategichie.com/patient-centered-data-home/ (accessed June 5, 2022).

