# Supplementary material for: Patient-Centered Data Home: A Path Towards National Interoperability
Source: Front Digit Health. 2022 Jul 13;4:887015. doi: 10.3389/fdgth.2022.887015 (PMC9328272; doi:10.3389/fdgth.2022.887015)
Supplement: Supplementary file 1 [file Data_Sheet_1.DOCX]

Supplementary Material


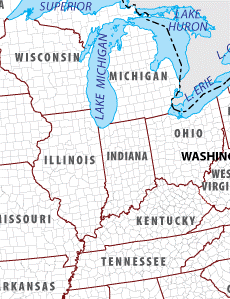


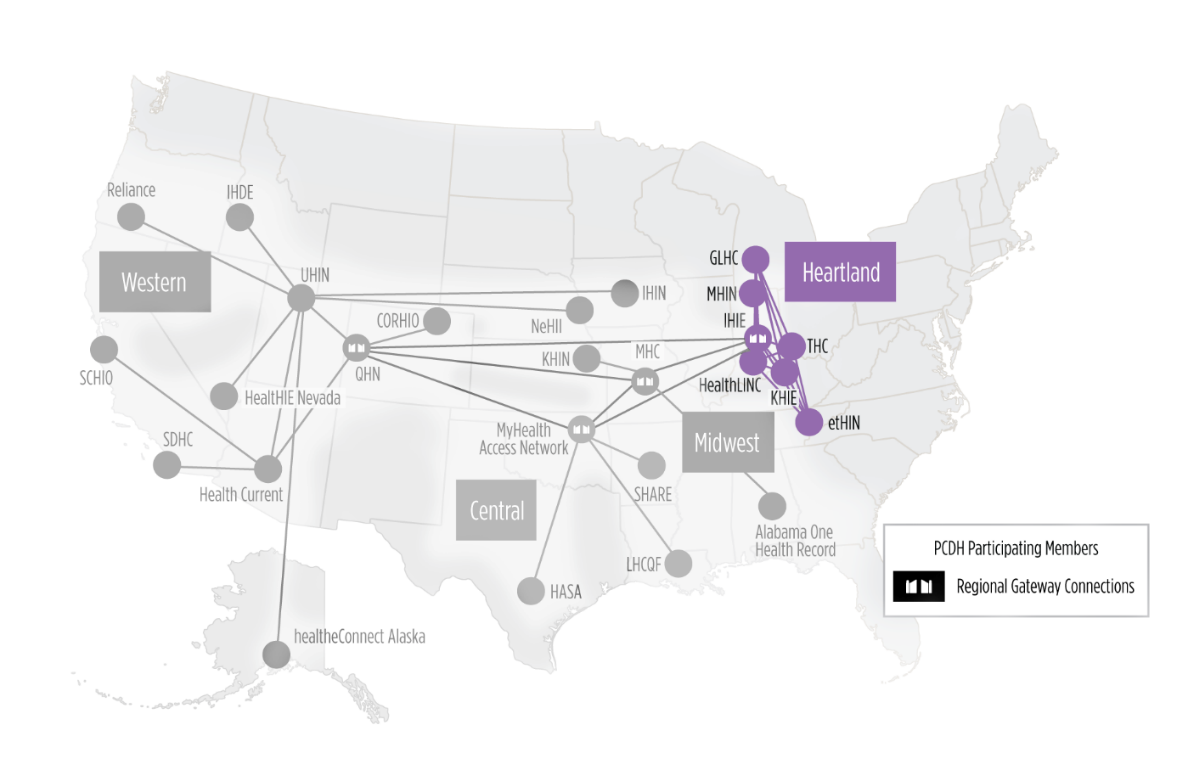


**Supplementary Figure 1.** Heartland Region of Patient-Centered Data Home


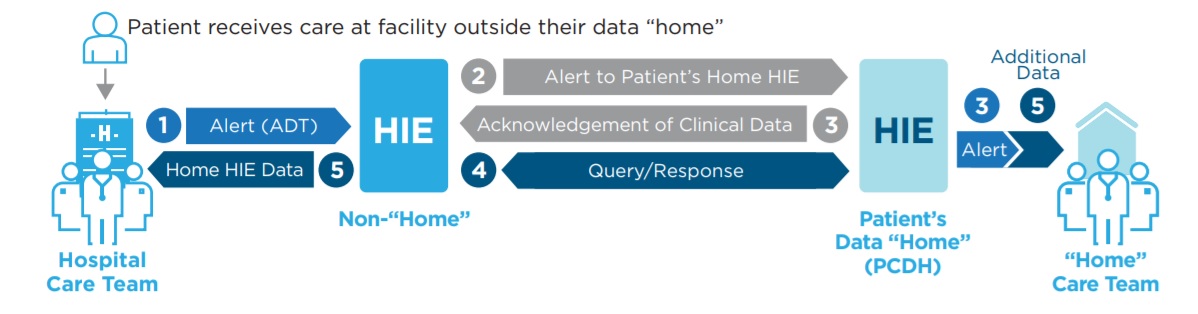
**Supplementary Figure 2.** Patient-Centered Data Home Process

**Supplementary Figure 3.** Methodology steps of PCDH study


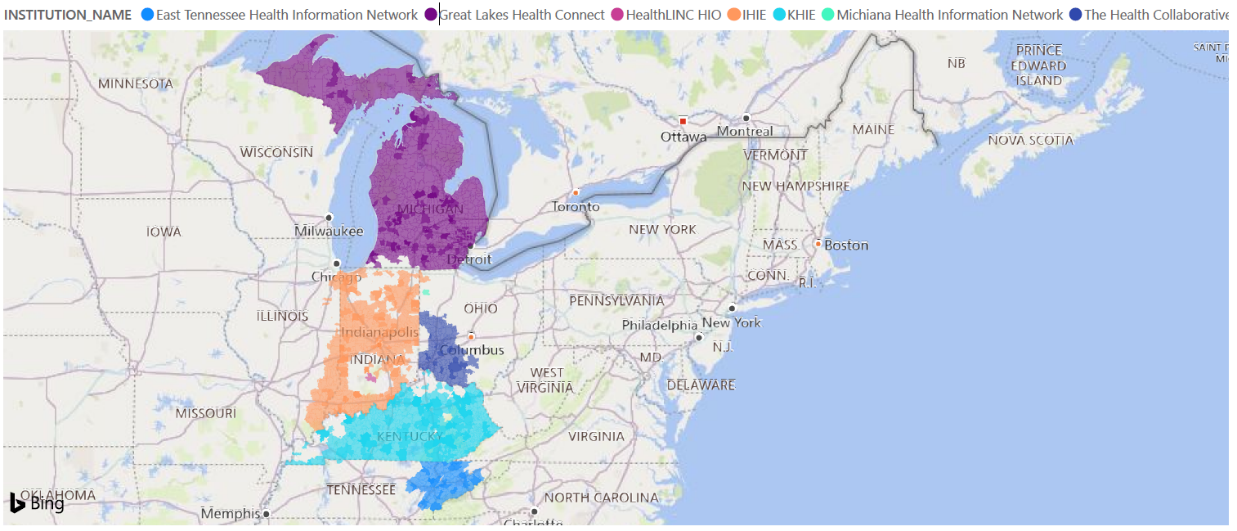


**Supplementary Figure 4.** Overlap Analysis of Heartland Region HIE Zip Codes

**Supplementary Table 1.** Unshared Zip Code counts and ADT messages within that zip code in the PCDH

| **Unshared PCDH Zip Codes** | | | |
| --- | --- | --- | --- |
| **HIEs** | **Number of Zip Codes** | **ADTs in Zip Code** | |
|  |  | **n** | **%** |
| Eastern Tennessee Health Information Network | 84 | 320 | 0.16% |
| Great Lakes Health Connect | 572 | 3172 | 1.55% |
| HealthLINC | 1 | 39 | 0.02% |
| Indiana Health Information Exchange | 700 | 181083 | 88.39% |
| Michiana Health Information Network | 394 | 15351 | 7.49% |
| Kentucky Health Information Exchange | 2 | 18 | 0.01% |
| The Health Collaborative | 190 | 4874 | 2.38% |
| TOTAL | 1943 | 204857 |  |

**Supplementary Table 2.** Shared Zip Code counts and ADT messages within that zip code in the PCDH

| **Shared PCDH Zip Codes** | | | |
| --- | --- | --- | --- |
| **HIEs** | **Number of Zip codes** | **% Overlap** | **ADTs in Zip Code** |
| Great Lakes Health Connect & Indiana Health Information Exchange | 2 | 0.5% | 870 |
| Great Lakes Health Connect & Indiana Health Information Exchange & Michiana Health Information Network | 5 | 1.3% | 5197 |
| Great Lakes Health Connect & Michiana Health Information Network | 17 | 4.3% | 1773 |
| HealthLINC & Indiana Health Information Exchange | 76 | 19.1% | 232499 |
| Indiana Health Information Exchange & Michiana Health Information Network | 106 | 26.7% | 673045 |
| Indiana Health Information Exchange & The Health Collaborative | 53 | 13.4% | 163087 |
| Indiana Health Information Exchange & Kentucky Health Information Exchange | 31 | 7.8% | 209535 |
| Indiana Health Information Exchange & Kentucky Health Information Exchange & The Health Collaborative | 31 | 7.8% | 340 |
| Kentucky Health Information Exchange & The Health Collaborative | 76 | 19.1% | 1131 |
| TOTAL | 397 |  | 1287477 |

**Supplementary Table 3.** Volume of ADT messages sent and received to the Indiana Health Information Exchange during the Heartland Region PCDH Pilot (December 2016 – December 2017)

| Heartland Region HIEs | RECEIVED by IHIE | SENT by IHIE |
| --- | --- | --- |
| Eastern Tennessee Health Information Network | 320 | 211 |
| Great Lakes Health Connect | 8473 | 7278 |
| HealthLINC | 162476 | 87694 |
| Kentucky Health Information Exchange | 79904 | 306735 |
| Michiana Health Information Network | 300444 | 372981 |
| The Health Collaborative | 134756 | 31095 |

**Supplementary Figure 5.** Age Categories by Percent of Exchange (n=1,492,109)

**Supplementary Table 4.** Deterministic Patient Matching Results

| Total records = 1,492,372 | SSN + DB + MB + YB | SSN + FN + MB + YB | SSN + LN + DB | SSN + LN + FN | LN + FN + G + DB + MB + YB | LN + FN + ADR + ZIP + YB | COMBINED |
| --- | --- | --- | --- | --- | --- | --- | --- |
| Matched record pairs total | 4,097,247 | 4,082,686 | 4,076,314 | 4,065,206 | 5,024,822 | 4,164,494 | 5,063,821 |
| Matched record pairs, *excluding duplicate encounters* | 4,078,595 | 4,064,152 | 4,057,774 | 4,046,748 | 5,001,807 | 4,145,573 | 5,040,537 |
| Matched Patient Groups | 208,159 | 208,039 | 207,943 | 207,898 | 271,121 | 258,921 | 271,299 |
| Records in at least one matched pair | 922,657 | 920,644 | 919,825 | 918,409 | 1,185,358 | 1,057,296 | 1,190,513 |
| % of Records in at least one matched pair | 61.8% | 61.7% | 61.6% | 61.5% | 79.4% | 70.8% | **79.8%** |
