# Supplementary material for: Patient-Centered Data Home: A Path Towards National Interoperability
Source: Front Digit Health. 2022 Jul 13;4:887015. doi: 10.3389/fdgth.2022.887015 (PMC9328272; doi:10.3389/fdgth.2022.887015)
Supplement: Supplementary file 2 [file Table_1.DOCX]

Supplementary Material

**Supplementary Table 1.** Unshared Zip Code counts and ADT messages within that zip code in the PCDH

| **Unshared PCDH Zip Codes** | | | |
| --- | --- | --- | --- |
| **HIEs** | **Number of Zip Codes** | **ADTs in Zip Code** | |
|  |  | **n** | **%** |
| Eastern Tennessee Health Information Network | 84 | 320 | 0.16% |
| Great Lakes Health Connect | 572 | 3172 | 1.55% |
| HealthLINC | 1 | 39 | 0.02% |
| Indiana Health Information Exchange | 700 | 181083 | 88.39% |
| Michiana Health Information Network | 394 | 15351 | 7.49% |
| Kentucky Health Information Exchange | 2 | 18 | 0.01% |
| The Health Collaborative | 190 | 4874 | 2.38% |
| TOTAL | 1943 | 204857 |  |
