# Supplementary material for: Patient-Centered Data Home: A Path Towards National Interoperability
Source: Front Digit Health. 2022 Jul 13;4:887015. doi: 10.3389/fdgth.2022.887015 (PMC9328272; doi:10.3389/fdgth.2022.887015)
Supplement: Supplementary file 3 [file Table_2.DOCX]

Supplementary Material

**Supplementary Table 2.** Shared Zip Code counts and ADT messages within that zip code in the PCDH

| **Shared PCDH Zip Codes** | | | |
| --- | --- | --- | --- |
| **HIEs** | **Number of Zip codes** | **% Overlap** | **ADTs in Zip Code** |
| Great Lakes Health Connect & Indiana Health Information Exchange | 2 | 0.5% | 870 |
| Great Lakes Health Connect & Indiana Health Information Exchange & Michiana Health Information Network | 5 | 1.3% | 5197 |
| Great Lakes Health Connect & Michiana Health Information Network | 17 | 4.3% | 1773 |
| HealthLINC & Indiana Health Information Exchange | 76 | 19.1% | 232499 |
| Indiana Health Information Exchange & Michiana Health Information Network | 106 | 26.7% | 673045 |
| Indiana Health Information Exchange & The Health Collaborative | 53 | 13.4% | 163087 |
| Indiana Health Information Exchange & Kentucky Health Information Exchange | 31 | 7.8% | 209535 |
| Indiana Health Information Exchange & Kentucky Health Information Exchange & The Health Collaborative | 31 | 7.8% | 340 |
| Kentucky Health Information Exchange & The Health Collaborative | 76 | 19.1% | 1131 |
| TOTAL | 397 |  | 1287477 |
