# Supplementary material for: Patient-Centered Data Home: A Path Towards National Interoperability
Source: Front Digit Health. 2022 Jul 13;4:887015. doi: 10.3389/fdgth.2022.887015 (PMC9328272; doi:10.3389/fdgth.2022.887015)
Supplement: Supplementary file 4 [file Table_3.DOCX]

**Table 3.** Volume of ADT messages sent and received to the Indiana Health Information Exchange during the Heartland Region PCDH Pilot (December 2016 – December 2017)

| Heartland Region HIEs | RECEIVED by IHIE | SENT by IHIE |
| --- | --- | --- |
| Eastern Tennessee Health Information Network | 320 | 211 |
| Great Lakes Health Connect | 8473 | 7278 |
| HealthLINC | 162476 | 87694 |
| Kentucky Health Information Exchange | 79904 | 306735 |
| Michiana Health Information Network | 300444 | 372981 |
| The Health Collaborative | 134756 | 31095 |
