# Supplementary material for: Patient-Centered Data Home: A Path Towards National Interoperability
Source: Front Digit Health. 2022 Jul 13;4:887015. doi: 10.3389/fdgth.2022.887015 (PMC9328272; doi:10.3389/fdgth.2022.887015)
Supplement: Supplementary file 5 [file Table_4.DOCX]

Supplementary Material

**Supplementary Table 4.** Deterministic Patient Matching Results

| Total records = 1,492,372 | SSN + DB + MB + YB | SSN + FN + MB + YB | SSN + LN + DB | SSN + LN + FN | LN + FN + G + DB + MB + YB | LN + FN + ADR + ZIP + YB | COMBINED |
| --- | --- | --- | --- | --- | --- | --- | --- |
| Matched record pairs total | 4,097,247 | 4,082,686 | 4,076,314 | 4,065,206 | 5,024,822 | 4,164,494 | 5,063,821 |
| Matched record pairs, *excluding duplicate encounters* | 4,078,595 | 4,064,152 | 4,057,774 | 4,046,748 | 5,001,807 | 4,145,573 | 5,040,537 |
| Matched Patient Groups | 208,159 | 208,039 | 207,943 | 207,898 | 271,121 | 258,921 | 271,299 |
| Records in at least one matched pair | 922,657 | 920,644 | 919,825 | 918,409 | 1,185,358 | 1,057,296 | 1,190,513 |
| % of Records in at least one matched pair | 61.8% | 61.7% | 61.6% | 61.5% | 79.4% | 70.8% | **79.8%** |
